# Postpartum Necrotizing Myositis of the Uterus

**DOI:** 10.1155/S1064744997000021

**Published:** 1997

**Authors:** Sebastian Faro

**Affiliations:** 1 Department of Obstetrics and Gynecology Rush Medical Center Chicago IL USA


Infectious Diseases in Obstetrics and Gynecology 5:2 (1997)
© 1997 Wiley-Liss, Inc.

### Images in Infectious Diseases in Obstetrics and Gynecology

*Section Editor: David E. Soper, M.D.*

### Postpartum Necrotizing Myositis of the Uterus

**Sebastian Faro, M.D., Ph.D.**
*Department of Obstetrics and Gynecology, Rush Medical Center, Chicago, IL*

### Legend to Image

Postpartum endometritis is often a mild to moderate infection which responds rapidly to broad spectrum antibiotics. Infrequently, a patient will develop diffuse thrombosis of the vasculature of the uterus, thereby decreasing tissue perfusion. This results in inadequate levels of antibiotic reaching the myometrium of the uterus. A polymicrobial infection can, therefore, become well established and develop into a synergistic necrotizing infection. Note in (**A**), the enlarged uterus, the gas present in the uterine myometrium, and necrosis of the lower segment. In (**B**), the gas pattern is diffusely noted throughout the myometrium. The markedly dilated bowel reflects the presence of intra-abdominal sepsis secondary to infected contents spilling from the disruption of the necrotic lower uterine segment. Antibiotic therapy, clindamycin plus gentamicin plus ampicillin, was ineffective because adequate tissue levels of antibiotics could neither be achieved nor sustained. A total abdominal hysterectomy was performed and the patient subsequently did well. © 1997 Wiley-Liss, Inc.

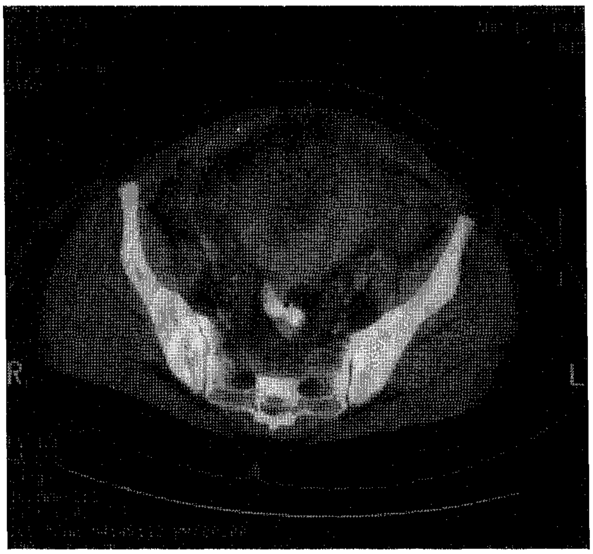

**A**

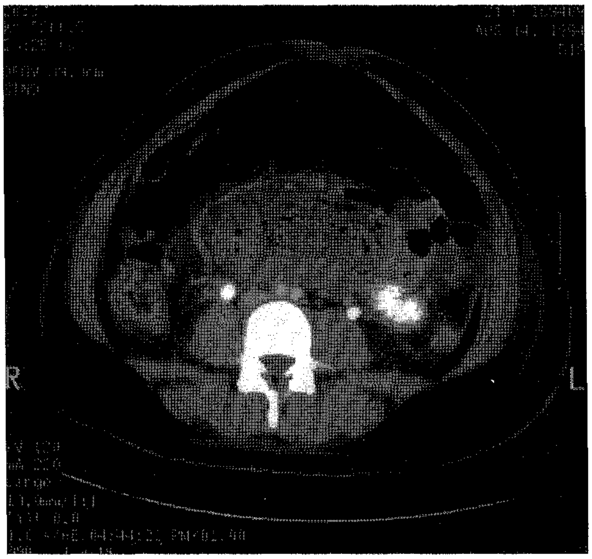

**B**

*Images in Infectious Diseases in Obstetrics and Gynecology* presents clinically important visual images that a practitioner in women's health might encounter. If you have a high-quality color or black-and-white photograph or slide representing such an image that you would like considered for publication, send it with a descriptive legend to David E. Soper, MD, Department of Obstetrics and Gynecology, Rush-Presbyterian-St. Luke's Medical Center, 1653 West Congress Parkway, Chicago, IL 60612-3833.
*Images* is made possible through an educational grant from Pfizer, Inc.

Received 15 May 1997
Accepted 15 June 1997

